# Artificial intelligence for oral squamous cell carcinoma detection based on oral photographs: A comprehensive literature review

**DOI:** 10.1002/cam4.6822

**Published:** 2024-01-02

**Authors:** Jérôme de Chauveron, Max Unger, Géraldine Lescaille, Laurent Wendling, Camille Kurtz, Juliette Rochefort

**Affiliations:** ^1^ Equipe Microenvironnement Immunitaire et Immunothérapie, CIMI Paris, INSERM U1135 Sorbonne université PARIS France; ^2^ UFR Mathématiques et Informatique, département informatique, laboratoire LIPADE Université Paris Cité Paris France; ^3^ ANALOGIES; ^4^ UFR d'Odontologie Université Paris Cité Paris France; ^5^ Département de Médecine bucco‐dentaire Hôpital Pitié Salpêtrière, APHP PARIS France

**Keywords:** artificial intelligence (AI), computer vision, diagnosis, diagnostic delay, head and neck carcinoma, Oral cancer, Oral squamous cell carcinoma

## Abstract

**Introduction:**

Oral squamous cell carcinoma (OSCC) presents a significant global health challenge. The integration of artificial intelligence (AI) and computer vision holds promise for the early detection of OSCC through the analysis of digitized oral photographs. This literature review explores the landscape of AI‐driven OSCC automatic detection, assessing both the performance and limitations of the current state of the art.

**Materials and Methods:**

An electronic search using several data base was conducted, and a systematic review performed in accordance with PRISMA guidelines (CRD42023441416).

**Results:**

Several studies have demonstrated remarkable results for this task, consistently achieving sensitivity rates exceeding 85% and accuracy rates surpassing 90%, often encompassing around 1000 images. The review scrutinizes these studies, shedding light on their methodologies, including the use of recent machine learning and pattern recognition approaches coupled with different supervision strategies. However, comparing the results from different papers is challenging due to variations in the datasets used.

**Discussion:**

Considering these findings, this review underscores the urgent need for more robust and reliable datasets in the field of OSCC detection. Furthermore, it highlights the potential of advanced techniques such as multi‐task learning, attention mechanisms, and ensemble learning as crucial tools in enhancing the accuracy and sensitivity of OSCC detection through oral photographs.

**Conclusion:**

These insights collectively emphasize the transformative impact of AI‐driven approaches on early OSCC diagnosis, with the potential to significantly improve patient outcomes and healthcare practices.

## Graphical abstract

This literature review offers an overview of the recent advancements in the application of artificial intelligence (AI) for oral cancer detection. All the studies exclusively employed oral photography for image data. Among the 11 articles, five achieved sensitivity rates exceeding 95%, while four attained accuracy rates surpassing 95%.

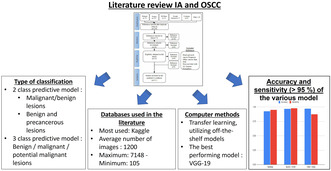

## 1 INTRODUCTION

As of 2018, upper aerodigestive tract cancers ranked as the sixth most prevalent cancer in France,^1^ and among them, oral squamous cell carcinomas (OSCC) constituted about a quarter of the cases. The primary risk factors for OSCC include alcohol and tobacco consumption,^2^ but these cancers can also develop in patients without these traditional risk factors, particularly in young individuals under 45 years of age,^2^ particularly affecting young women with tongue cancer.^3^ For these patients, the causes of OSCC remain unidentified but unlike oropharyngeal carcinomas^3^ there is limited evidence linking human papillomavirus (HPV) to OSCC, although some studies contradict this association.^4^, ^5^, ^6^

Oral squamous cell carcinoma (OSCC) is associated with a high annual mortality rate of 170,000 cases, primarily due to late‐stage diagnosis, resulting in a low 5‐year survival rate. The median age at which OSCC is typically diagnosed is 66 for women and 62 for men.^7^ Early detection and screening play a pivotal role in improving patient prognosis, as survival rates are closely tied to the stage of cancer at the time of diagnosis. Artificial intelligence (AI) has the potential to offer innovative diagnostic tools that can facilitate early detection.^8^

In recent years, the convergence of computer vision and artificial intelligence (AI) has sparked a paradigm shift in medical diagnostics. Specifically, the application of these technologies to the detection of oral cancer from digitized oral photographs holds tremendous promise for revolutionizing the accuracy and timeliness of diagnosis. Oral cancer, a significant global health challenge, often evades early detection, resulting in advanced stages and reduced survival rates. However, with the advent of sophisticated image analysis techniques and (deep) machine learning algorithms such as convolutional neural networks (CNN), the field of oral cancer detection is undergoing a profound transformation.

Remarkably, one of the significant advancements in oral cancer detection involves the application of deep learning for the classification of histopathological images^9^, ^10^ achieving an accuracy of 96.6% and 98.4%, comparable to the performance of a specialist doctor. It is important to note that this literature review will exclusively concentrate on the detection of oral cancer using photographic images and will not delve into the detection utilizing histopathological images.

This literature review critically examines the existing body of research that harnesses the power of computer vision and AI to enhance oral cancer detection. By synthesizing and analyzing key studies, methodologies, and outcomes, this paper aims to provide a comprehensive overview of the advancements in the intersection of medical and computer sciences. Through this exploration, we aim to elucidate the evolving role of machine vision in reshaping the future of oral cancer diagnosis.

### 1.1 Introduction and definitions related to Machine Learning and Computer Vision

Neural networks are a fascinating breed of machine learning algorithms, drawing inspiration from the intricate workings of the human brain. Interconnected nodes within these networks, each performing simple mathematical operations, collectively grasp patterns from copious data provided during the training phase.

An essential training algorithm, Backpropagation, becomes the driving force behind empowering neural networks. Through meticulous error calculation and systematic weight adjustments based on the network's output, it refines the model's capabilities. This iterative process culminates when the neural network masters generating precise outputs for various inputs.

In the context of image analysis, CNNs play a vital role. CNNs are a class of neural networks specifically tailored for processing grid‐like data, such as images. They utilize specialized layers to automatically and adaptively extract features from input images. This hierarchical feature extraction is instrumental in identifying complex patterns in medical imagery, aiding in accurate cancer detection and analysis.

One notable advantage of CNNs lies in their capacity to extract image features and generalize across various computer vision problems. Nevertheless, their efficacy relies on substantial labeled data, and their computational demands are considerable.

Additionally, transfer learning is a powerful strategy in neural network training. It involves using knowledge gained from training one model on a specific task and applying it to a related task. This approach can significantly speed up training and improve performance, especially when datasets for the new task are limited.

Furthermore, attention mechanisms, mimic cognitive attention, they select and focusing on pertinent aspects of the input. They elevate the critical elements while subtly fading out the rest, like human attention focusing on salient details. However, attention mechanisms are still relatively recent in the field of medical imaging, demanding a substantial amount of training data and being highly computationally intensive.

Finally, within image analysis tasks, we encounter crucial tasks: classification involves identifying the type of object within an image by predicting a semantic label, detection encompasses pinpointing the presence and position of objects in an image, and segmentation entails identifying pixels associated with each object in the image. Each task adds a layer of sophistication to the network's comprehension and utilization of intricate data structures.

## 2 MATERIAL AND METHODS

A systematic review was conducted between May 2023 and September 2023. The research protocol was registered on the International Prospective Register of Systematic Reviews PROSPERO on number CRD42023441416.

### 2.1 Objectives

The primary objective was to assess the performance of the employed AI models in terms of accuracy and F1 score, to examine the dataset regarding its size and medical validity, and to evaluate the effectiveness of preprocessing and cleaning techniques (usually employed in computer vision applications), as well as the machine learning methods employed. Subsequently, we aimed to identify the most promising and intriguing approaches for oral cancer detection, taking into consideration dataset dimensions.

The secondary objective was to highlight the limitation of the current research in this field including issues related to both the quality and size of the dataset employed and to discuss potential enhancements for machine learning approaches such as data augmentation, choice of model / supervision strategies, and the utilization of ensemble learning.

### 2.2 Search strategy

**FIGURE 1:**
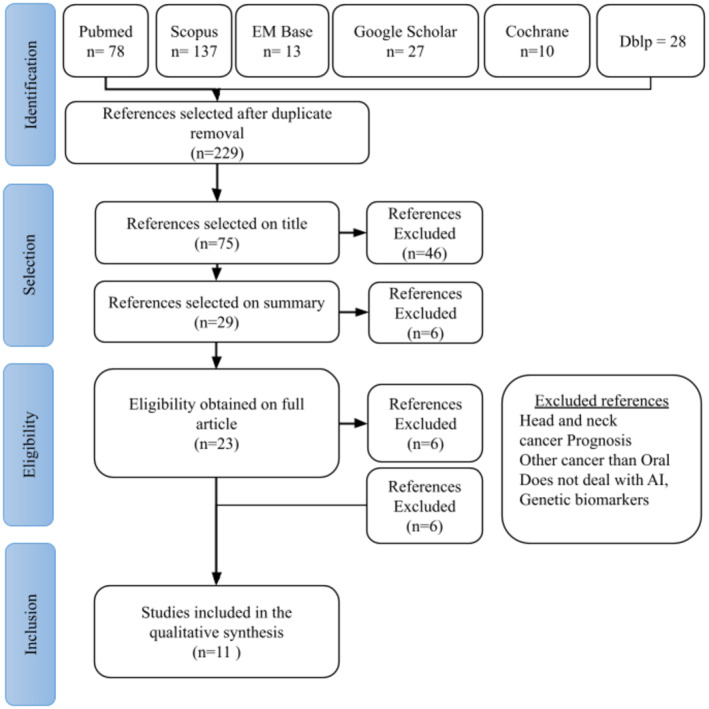
PRISMA flow diagram of the literature search. First step of identification using keywords on various databases and removal of duplicates; second step of selection based on title and then on abstracts; third step of eligibility after complete reading of articles and analysis of their methodology; fourth step of inclusion.

This literature review followed the scoping review methodology outlined in the Joanna Briggs Institute guide, and its methodology adhered to PRISMA guidelines (Figure 1). The research question driving this review was as follows: Can artificial intelligence effectively serve as a tool for detecting and diagnosing oral cavity cancers based through the analysis of oral photographs from patients?

#### 2.2.1 Choice of keywords used

The terms used during the search were as follows: Mouth Neoplasm (Mesh term), Oral Squamous cell carcinoma, OSCC, Oral cancer, Computer vision, Machine learning, Deep Learning, and CNN.

#### 2.2.2 Database used

We have used the following databases: Pubmed, Cochrane Scopus, Embase, Dentistry and oral science source, dblp, Arxiv, and Google scholar. The same research was performed on the gray literature (OpenGrey and Google Scholar).

For the Pubmed database, the equation performed was as follows: ((((ai artificial intelligence[MeSH Terms]) OR (machine learning[MeSH Terms]) OR (deep learning[MeSH Terms]) OR (diagnosis, computer‐assisted[MeSH Terms]) OR (Artificial intelligence) OR (deep learning) OR (machine learning) OR (diagnosis, computer‐assisted)))) AND (((mouth neoplasms) OR (oral cancer) OR (oral squamous cell carcinomas) OR (mucosal lesions) OR (mouth neoplasms[MeSH Terms]) OR (oral cancer[MeSH Terms]) OR (oral squamous cell carcinomas[MeSH Terms]))) AND (y_5[Filter]) AND ((computer vision) OR (vision) OR (CNN)).

For the Embase database, the equation performed was as follows: (‘artificial intelligence’/exp OR ‘artificial intelligence’) AND (‘mouth cancer’/exp OR ‘mouth cancer’) AND [2017–2022]/py.

For the Scopus database, the equation performed was as follows: (TITLE‐ABS‐KEY (artificial AND intelligence) OR TITLE‐ABS‐KEY (machine AND learning) OR TITLE‐ABS‐KEY (deep AND learning) AND TITLE‐ABS‐KEY (oral AND cancer) OR TITLE‐ABS‐KEY (oral AND squamous AND cell AND carcinoma) OR TITLE‐ABS‐KEY (mouth AND neoplasms)) AND (LIMIT‐TO (PUBYEAR, 2022) OR LIMIT‐TO (PUBYEAR, 2021) OR LIMIT‐TO (PUBYEAR, 2020) OR LIMIT‐TO (PUBYEAR, 2019) OR LIMIT‐TO (PUBYEAR, 2018) OR LIMIT‐TO (PUBYEAR, 2017)).

For the Web Of Science database, the equation performed was as follows: ((ALL = (oral cancer) OR ALL = (OSCC)) AND (ALL = (computer vision) OR ALL = (CNN)) AND (PY==(“2017” OR “2018” OR “2019” OR “2020” OR “2021” OR “2022” OR “2023”))).

For the Google Scholar database, the equation performed was as follows: “artificial intelligence” “oral cancer” “photography” “computer vision”.

For the dblp database, the equation performed was as follows: oral cancer detection|recognition.

Two authors have independently extracted data and consulted the third author when needed.

### 2.3 Definition of variables

Oral squamous cell carcinoma (OSCC), which stands for the uncontrolled and chaotic proliferation of epithelial cells, is characterized by their rapid expansion leading to local tissue destruction. Depending on the stage, OSCC may or may not be associated with distant dissemination, and the formation of metastasis. Epidermoid carcinoma accounts for 90% of malignant tumors in the oral cavity.

The artificial intelligence (AI) encompasses a wide range of techniques and technologies aim at creating systems capable of mimicking human‐like cognitive functions (reasoning, problem solving, perception, and language understanding).

The computer vision, a subfield of AI, focuses on enabling machines to interpret and comprehend visual information from the world. It involves the development of algorithms and techniques that empower computers to analyze, process, and make sense of images or videos.

### 2.4 Inclusion criteria

In order to be included, articles were required to have a primary focus on OSCC detection from oral photographs using AI tools. Studies were eligible for inclusion if they had been published within the past 5 years. No language restrictions were imposed as long as translations were available. As a result, we included both experimental and observational studies.

### 2.5 Exclusion criteria

We excluded studies involving animal or in vitro models from our selection. Duplicate entries within the same database were eliminated. Articles that did not primarily address the detection of epidermoid carcinoma through the utilization of artificial intelligence tools or oral photographs were excluded.

### 2.6 Data extraction

After eliminating duplicates, two independent investigators (JDC and JR) reviewed titles and abstract to select articles. Both review authors independently extracted relevant articles initially based on title, and subsequently, they considered abstract and full‐text papers. To ensure comprehensiveness, the bibliographic sources of the selected articles were examined to include any additional articles that our search may have missed. The authors extracted details regarding study design, participant characteristics, intervention and comparator, and outcomes. The research was conducted using the software tools Zotero and Rayyan.

### 2.7 Quality assessment

The authors have graduated the levels of evidence of the articles obtained (Table 1). With the help of the HAS literature analysis guide.

**TABLE 1:** Analysis of quality assessment using HAS grading.

Study	HAS level of proof
Nanditha B R et al 2021^11^	Intermediate; Grade 4C
Gizem Tanriver et al 2021^12^	Intermediate; Grade 4C
Mohammed Zubair M. Shamim et al 2022^13^	Intermediate; Grade 4C
Zhiyun Xue et al 2022^14^	Intermediate; Grade 4C
Roshan A. Welikala 2021^15^	Intermediate; Grade 4C
Roshan Alex Welikala et al 2020^16^	Intermediate; Grade 4C
Adwan A. Alanazi et al 2022^17^	Intermediate; Grade 4C
Huan Ding et al 2023^18^	Intermediate; Grade 4C
Qirui Huang et al 2023^19^	Intermediate; Grade 4C
Kamil Jurczyszyn et al 2020^20^	Intermediate; Grade 4C
Qiuyun Fu et al 2020^21^	Intermediate; Grade 4C

## 3 RESULTS

### 3.1 Literature search description

#### 3.1.1 Selected articles

After completing the literature search, we acquired 78 articles from the PubMed database, 137 from the Scopus transdisciplinary database, 13 on EMBASE, and 75 from Web of Science. Additionally, we found 27 articles on Google Scholar 28 on dblp. The Cochrane database did not permit the inclusion of articles on this topic. It is worth noting that since arxiv is an open database where publishing doesn't require peer review, all the paper from arxiv were also present in the other database. The study finally included 11 articles (see Figure 1).

#### 3.1.2 Type of articles included

Among the articles included in our study, none of them were prospective or retrospective cohort studies, 11 were diagnostic studies, and none were case reports or literature reviews.

#### 3.1.3 Level of proof of the articles

All these analyzed papers listed in Table 1, presented an intermediate level of proof of according to the HAS.

#### 3.1.4 Extraction of clinical data

For each article, we recorded the following information: author, title, type of study, and year of publication (refer to Table 2). Furthermore, we collected details regarding the database of digitized oral photographs employed, including its size, quality, and validity; the controls implemented within the study; the AI tools utilized; and the achieved detection performance (accuracy, sensitivity and specificity). These data have been documented in Table 2.

**TABLE 2:** Data from the studies included in the systematic review.

Author/Year of publication	Type, number and validity of data base	Type of controls used	Model type	Performance	Sensitivity	Specificity
Nanditha B R et al 2021^11^	332 images (63 benign, 269 precancerous)	Benign, Precancerous	ResNet50 VGG‐Skip	96.2% Accuracy	98.14%	94.23%
Gizem Tanriver et al 2021^12^	162 lesions annotated by an expert oral pathologist	Benign, Malignant OPMD	EffiscientNetb4	87% Accuracy	86%	NC
Mohammed Zubair M. Shamim et al 2022^13^	200: binary classification 300: multi‐class (OPMD) annotated by a certified physician	Benign, Malignant	VGG19	98% Accuracy	89%	97%
Zhiyun Xue et al 2022^14^	7148 (no histopathologic proof mentioned)	Benign, Malignant	Resnet	98% Accuracy	100%	99.6%
Roshan A. Welikala 2021^15^	2155 annotated by 3–7 experts clinicians	Benign, Malignant	VGG With clinically guided attention	83.3% Accuracy	NC	NC
Roshan Alex Welikala et al 2020^16^	1180 (part of the MeMoSa project)	Benign, Malignant OPMD	VGG19	80.88% Accuracy	85.71%	76.42%
Adwan A. Alanazi et al 2022^17^	105 (kaggle dataset^22^)	Benign, Malignant	NASNet	92.59% (training 80: test:20)	95% (training 80; test:20)	NC
Huan Ding et al 2023^18^	105 (kaggle dataset^22^)	Benign, Malignant	MLSO + SVM	96.94%	97.70%	92.37%
Qirui Huang et al 2023^19^	105 (kaggle dataset^22^)	Benign, Malignant	CNN + ISSA	97.33%	87.34%	NC
Kamil Jurczyszyn et al 2020^20^	35 patients with leucoplakia, Histopathologic diagnosis 35 patients with normal oral mucosa	Cancer, Normal Mucosa	Probabilistic neural network	NC	100%	97%
Qiuyun Fu et al 2020^21^	1469 With histopathologic diagnosis	Benign, Malignant	CNN	92.3%	91.0%	93.5%

### 3.2 Summary of results

#### 3.2.1 Databases of digitized oral photographs used

Among all the papers we analyzed, three of them employed an open dataset from Kaggle for benchmarking purposes.^22^ This database contains 87 images labeled as “malignant lesions,” with no diagnostic evidence. There is no information about the type of device used to take those pictures, and they are of different sizes and fairly large with an average size of 769 by 601 pixels. After review by photo‐interpretation by our expert (JR), it was found that some of these lesions were not representative of OSCC. The average dataset size across these studies is approximately 1200 images, with the largest dataset containing 7148 images^14^ and the smallest containing only 105 images.^17^, ^18^, ^19^ Moreover, no information is given on the size or device used to take those images. Although these dataset sizes may not match the scale of some other computer vision research areas, the results achieved are promising, even though there could be overfitting (the model fitting too close to the training data attribute instead of real‐world characteristics) due to the limited amount of data.

#### 3.2.2 Type of classification

Among all the research papers considered, all but two utilized a two‐class predictive model. 7 out the 11 papers distinguished only malignant or benign lesions,^13^, ^14^, ^15^, ^17^, ^18^, ^19^, ^21^ one distinguished malignant and healthy thy tissues,^20^ one distinguished Benin and precancerous lesions,^11^ and two of them have a third‐class: potential malignant lesions.^12^, ^16^

#### 3.2.3 Computer methods

All the research papers employed transfer learning, utilizing off‐the‐shelf models (already existing model that were re‐trained on oral cancer images with transfer learning). The best performing model among them was VGG‐19.^11^, ^13^, ^15^, ^16^ VGG is one of simpler type of CNN and can generalize well to different tasks. Resnet utilize skip connection (the output from one layer bypass one or more intermediate layers and is directly added to the output of a later layer, helping to mitigate the vanishing gradient problem and facilitate the training of very deep networks) it is a more complex model however it requires more data consequently it is not the best suited model architecture for this particular task.

EffiscientNet is also a more advanced architecture utilizing scaling method and being one of the model requiring the least computing power. It is also not the best fitted model for OSCC detection as it doesn't generalize well to new detection task and computing power isn't a pressing issue as dataset are not very large.

In summary, the most promising machine learning approaches used by authors are as follows:

- Texture descriptor: These features can be extracted from images to describe the spatial arrangement of pixels. They have demonstrated effectiveness in classifying oral cancer cells, by capturing subtle changes in texture associated with cancer^20^;
- Attention model: These neural networks can learn to focus on specific regions of an image. This ability can be helpful for of oral cancer detection by allowing the model to disregard irrelevant information and concentrate most critical features for classification^15^;
- Localization with multi‐task learning: Two different machine learning techniques, they are used jointly for the scope of this study. Localization involves identifying the location of a specific object in an image. Multi‐task learning, a machine learning technique, enables a model to learn multiple tasks simultaneously. Those two techniques can be used separately but it was found that their combined usage is promising^23^;
- Ensemble approach: This technique combines predictions from multiple models to enhance overall performance. It helps reduce the risk of overfitting and enhances the model's robustness in oral cancer detection^11^;
- Data augmentation artificially increases the dataset's size by generating new data from existing samples, improving the model's ability to generalize and detect oral cancer more accurately.^19^

#### 3.2.4 Accuracy and sensitivity of the various model types

**FIGURE 2:**
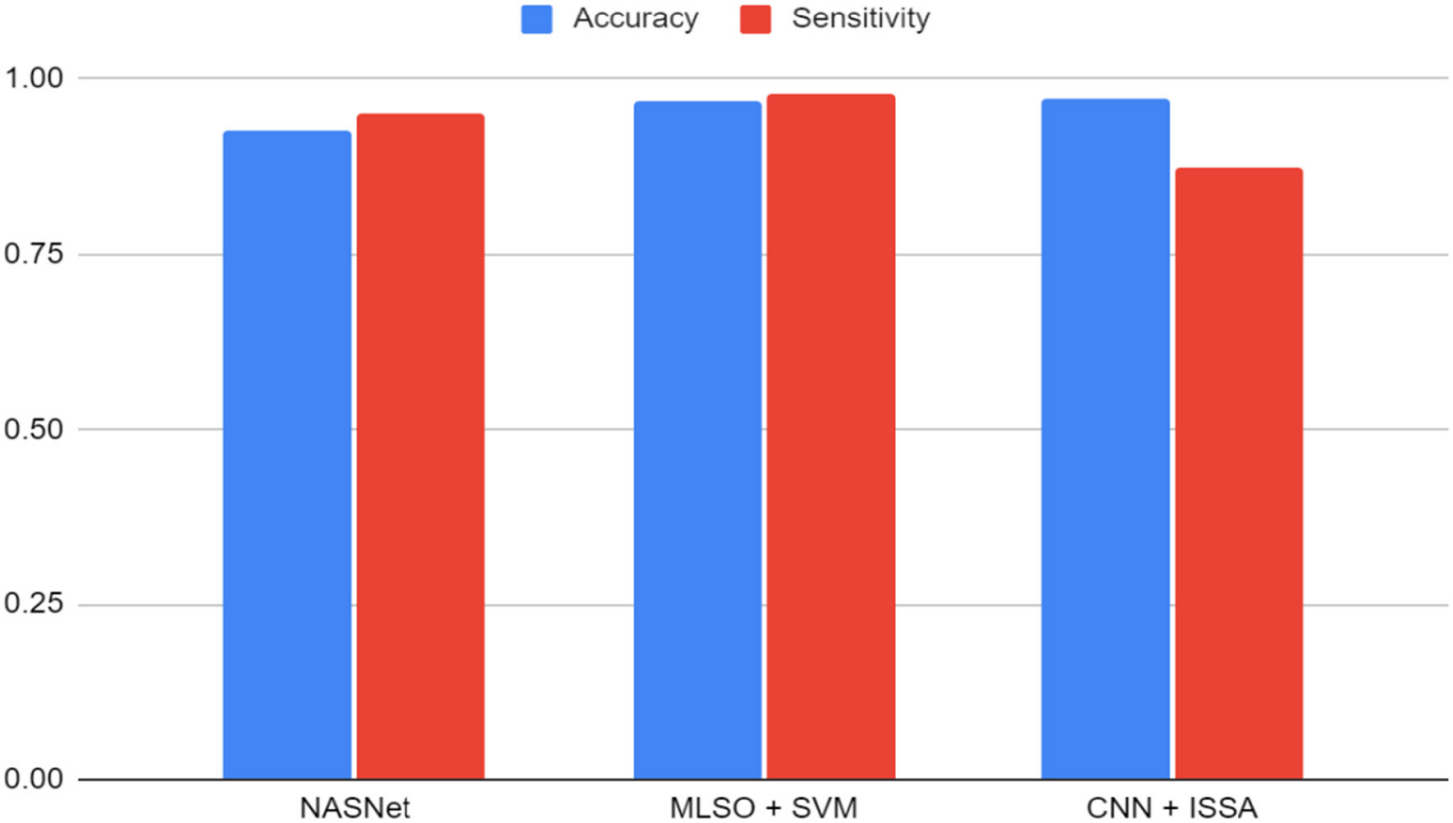
Accuracy and sensitivity for different type of models for the oral lesion recognition task (models benchmarked on the Kaggle dataset [11]).

Finally, in Figure 2 below, we present the accuracy and sensitivity of the various model types used in these studies. It is very difficult to compare those performances as they were not obtained on the same dataset: not the same number of images and not the same quality. In this context, we only compared the results obtained on the same dataset from Kaggle.^22^ In this comparison, MLSO + SVM^18^ has significantly better performances than the other models. It is worth noting that despite being trained with a substantial dataset of 2155 images,^16^ VGG with attention^16^ emerged as one of the worst‐performing models.

#### 3.2.5 Results achieved in terms of cancer detection capability

Furthermore, five of the papers under study achieved sensitivity rates above 95%,^11^, ^14^, ^17^, ^18^, ^20^ with four of them relying on training datasets of fewer than 500 images^11^, ^17^, ^18^, ^20^ and only one of these papers used a dataset larger than 1000 images.^14^

It is important to note that sensitivity is the most crucial metric in our case, as a false negative result (when the model predicts benign lesion when it is malignant) can have severe consequences.

#### 3.2.6 Factors influencing the tool's detection performance

**FIGURE 3:**
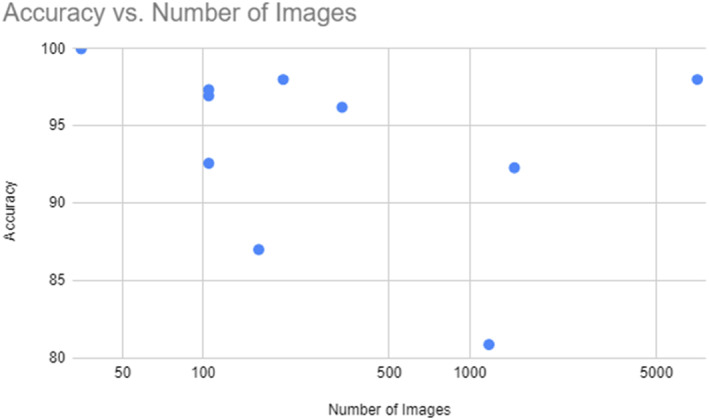
Models’ accuracy versus number of training images.

**FIGURE 4:**
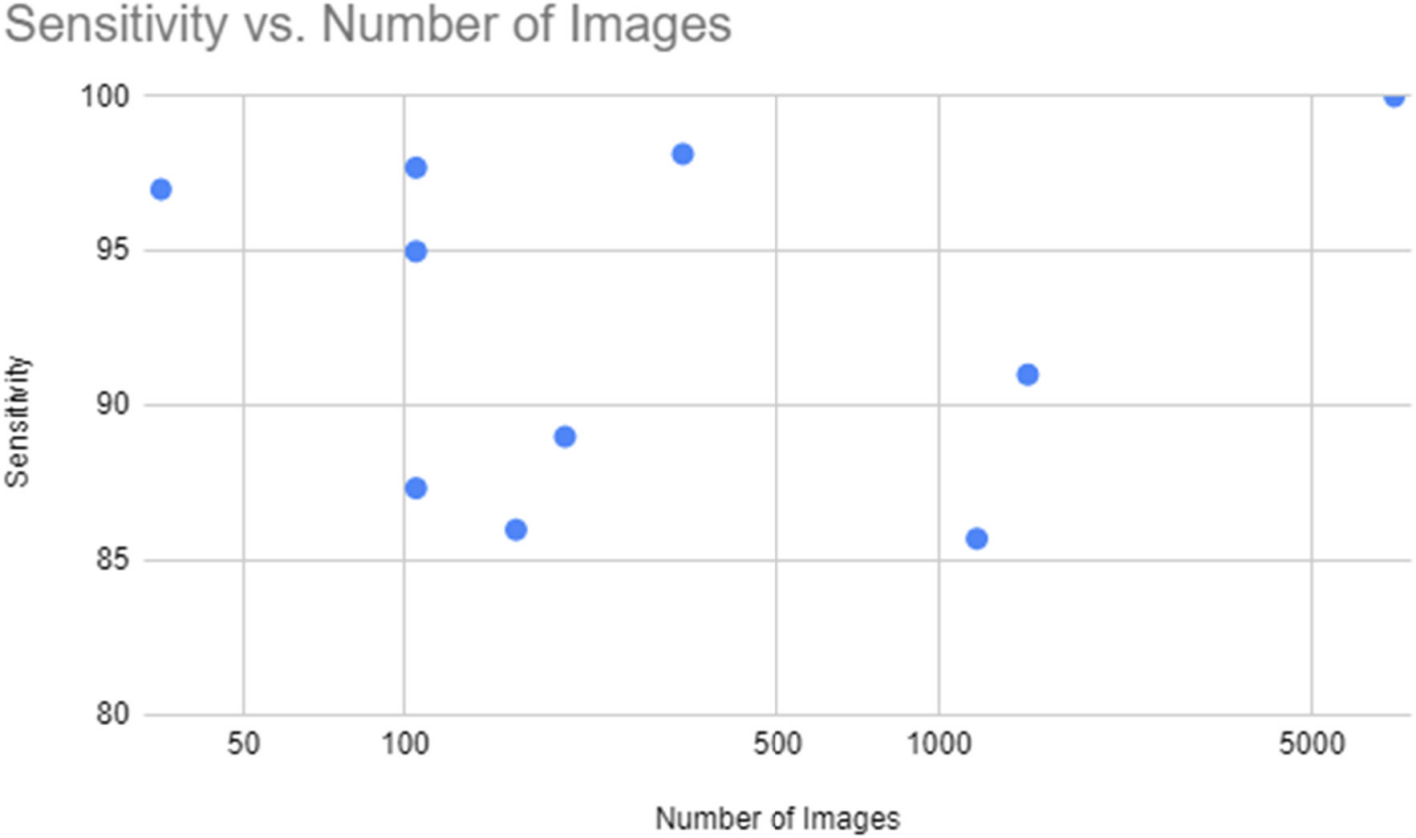
Model sensitivity versus number of training images.

As demonstrated in the following Figures 3 and 4, we can see that, for this specific study, the number of images did not exert a large influence on the accuracy or sensitivity of the models. However the quality of those dataset is not necessary always the same which makes the comparison more difficult. This is particularly noteworthy when considering models that incorporate more than a convolutional network, such as MLSO, ISSA, and the probabilistic neural network as they performed well with little data.

The number of classes has a significant influence as the two models detecting malignant, Benign and OPMD lesions were not the best performing models.^12^, ^16^

## 4 DISCUSSION

### 4.1 Encouraging results for oral cancer detection using computer vision

The application of computer vision in oral cancer detection has yielded highly promising results, showcasing encouraging results (with limited available data): accuracy levels of up to 99% and F1 scores reaching 94.9%.^11^, ^14^, ^17^, ^18^, ^20^ These achievements are particularly impressive considering the relatively modest dataset sizes, with an average of 1200 images per study,^11^, ^12^, ^13^, ^14^, ^15^, ^16^, ^17^, ^18^, ^19^, ^20^, ^21^ and the largest dataset containing 7148 image,^14^ while the smallest comprised only 105 images.^17^, ^18^, ^19^ It is crucial to acknowledge that collecting data for medical images, particularly in the context of OSCC, is inherently more labor‐intensive and resource demanding.

### 4.2 Promising machine learning approaches

Several key strategies have emerged to enhance performance in oral cancer detection. The introduction of attention mechanisms, although still in its early stages of exploration, demonstrates its potential as a viable approach. A recent study^15^ incorporating attention mechanisms showcased the novel possibilities that arise when focusing computational resources on relevant image regions, significantly improving detection capabilities. Moreover, new research^24^ shows that attention models / transformers (which typically require large amount of data) can also be trained on a relatively small dataset. Ensemble learning, too, has exhibited significant promise.^11^ A notable example achieved a remarkable accuracy rate of 96.2%,^11^ highlighting the synergistic effects of combining multiple models for enhanced classification. This approach capitalizes on the diversity of individual models to collectively achieve robust predictions, even with smaller datasets. Image enhancement techniques have emerged as a noteworthy avenue for boosting detection accuracy. Despite their application on relatively smaller datasets, these techniques have proven effective in refining image quality, leading to improved model performance.^11^

The integration of landmark detection^23^ represents an intriguing approach that utilizes anatomical landmarks to guide the detection process. Leveraging multi‐task learning enhances the precision of cancer detection, holding the potential for more accurate and targeted diagnoses. Among the architecture choices, VGG‐19 emerges as a standout performer, consistently achieving the best results across multiple studies. Its ability to extract complex features from images aligns well with the demands of oral cancer detection, contributing to the overall high accuracy rates observed.

### 4.3 Dataset limitations and future directions

However, the available dataset for this purpose is rather limited.^25^ Furthermore, the reference benchmark dataset for oral cancer detection is relatively small and lacks verification through biopsy diagnosis. Therefore, data augmentation and preprocessing are of utmost importance. Employing techniques such as image enhancement through discrete wavelet transform and adaptive histogram equalization^18^ offers an intriguing approach. Even with standard data augmentation techniques like rotation, flipping, and cropping, it is clear that there is substantial room for improvement and further research. One notable constraint frequently observed is the absence of standardization in the utilized datasets, which makes comparing different studies more challenging. The most commonly employed benchmark dataset, sourced from Kaggle,^22^ has exhibited reliability issues, and is relatively small. As a result, the findings from studies using this dataset may not readily apply to diverse settings.

While employing computer vision for oral cancer detection holds promise, addressing the potential for false positives and false negatives is paramount. False positives can induce unnecessary anxiety and follow‐up procedures, while false negatives may impede timely diagnosis and treatment, posing potential life‐threatening risks for the patient. One effective strategy to mitigate these risks involves incorporating a follow‐up assessment to evaluate the evolution of lesions. Additionally, implementing other strategies such as robustness testing (involving a diverse range of lesions, lighting conditions, resolutions, etc.) and human‐in‐the‐loop confirmation by an expert when the model is uncertain about the pathology can significantly enhance the accuracy and reliability of computer vision models.

Despite these limitations, the findings from this literature review underscore the potential of AI as a valuable tool for early oral cancer detection. Further research is imperative to construct and validate AI‐based systems tailored for oral cancer detection. This future research should prioritize the use of larger and more dependable datasets and the development of AI models capable of withstanding variations in imaging conditions.

## 5 CONCLUSION

This literature review offers an overview of the recent advancements in the application of artificial intelligence (AI) for oral cancer detection. The findings from the studies included in this review underscore the potential of AI as a valuable tool for early oral cancer detection, with the promise of enhancing patient outcomes.

The reviewed studies employed a range of AI techniques, encompassing machine learning, deep learning, and computer vision. Among the machine learning algorithms, convolutional neural networks (CNN) were the most frequently used. Some studies also incorporated support vector machines (SVM) and probabilistic neural networks.

All the studies exclusively employed oral photography for image data. Among the 11 articles, five achieved sensitivity rates exceeding 95%, while four attained accuracy rates surpassing 95%. Interestingly, the quantity of images does not appear to be a limiting factor, and the utilization of texture descriptors, ensemble learning, and multi‐task learning approaches has proven to be highly effective.

In addition to the points discussed earlier, here are some other noteworthy findings from the literature review:

- The application of AI in oral cancer detection is still in its nascent stages, but the results from the reviewed studies show promise.
- AI holds the potential to enhance the accuracy and efficiency of oral cancer detection, ultimately leading to earlier diagnoses and improved patient survival rate.

The insights from this literature review suggest that the most promising approaches either involve combining CNN with another method or exclude the use of CNN altogether. This is partly attributed to the limited size of the dataset, elucidating why the lone attention‐based model performs poorly (attention models typically demand a larger dataset). However, advancements in techniques enabling the training of attention models with fewer data may alter this landscape.^26^

Computer vision is revolutionizing the detection and management of cancers. From the perspective of healthcare professionals, this advancement has a significant impact on clinical practice. Indeed, computer vision enables the detection of cancer signs in medical images, such as X‐rays, MRIs, CT scans, mammograms, and even photographs. It can identify anomalies that sometimes elude the human eye. This ability to identify lesions at an early stage has a major impact on the patient's prognosis, allowing for rapid intervention and targeted treatment. The impact of computer vision on cancer detection goes beyond information technology. It transforms how healthcare professionals diagnose and treat patients, offering significant benefits such as detection accuracy, reduction of human errors (by providing an objective and reproducible analysis of medical data), workload relief (by automating routine tasks like lesion detection), and access to global expertise through the exchange of digital data. This medical revolution represents a significant step toward improving outcomes for cancer patients.

## AUTHOR CONTRIBUTIONS

**Jérôme de chauveron:** Data curation (equal); formal analysis (equal); investigation (equal); writing – original draft (equal). **Max Unger:** Project administration (equal); validation (equal); writing – review and editing (equal). **Géraldine Lescaille:** Project administration (equal); validation (equal); writing – review and editing (equal). **Laurent Wendling:** Methodology (equal); project administration (equal); validation (equal); writing – review and editing (equal). **Camille Kurtz:** Formal analysis (equal); methodology (equal); project administration (equal); validation (equal); writing – original draft (equal); writing – review and editing (equal). **Juliette Rochefort:** Conceptualization (equal); formal analysis (equal); funding acquisition (equal); methodology (equal); project administration (equal); validation (equal); writing – original draft (equal); writing – review and editing (equal).

## CONFLICT OF INTEREST STATEMENT

The authors have no conflict of interest to declare. These statements emphasize our dedication to transparency and ethical conduct in research. It is crucial to uphold stringent ethical standards, particularly when data availability is limited, to ensure the accuracy and reliability of the findings presented in this literature review.

## Data Availability

This literature review on oral cancer detection extensively examines studies from various sources. While efforts were made to access datasets, most referenced datasets were not made accessible by the original authors. Notably, one dataset from Kaggle https://www.kaggle.com/code/shivam17299/oral‐cancer‐lips‐and‐tongue‐images‐dataset was accessible and referenced in this review. Due to limitations in data accessibility imposed by original authors, the majority of referenced datasets remain unavailable. The Kaggle dataset used is acknowledged for its contribution. All utilized datasets are appropriately cited in the bibliography for transparency and reference. For further details on the referenced datasets, we recommend contacting the original authors or publishers.
